# The Pleiotropic CymR Regulator of *Staphylococcus aureus* Plays an Important Role in Virulence and Stress Response

**DOI:** 10.1371/journal.ppat.1000894

**Published:** 2010-05-13

**Authors:** Olga Soutourina, Sarah Dubrac, Olivier Poupel, Tarek Msadek, Isabelle Martin-Verstraete

**Affiliations:** 1 Institut Pasteur, Unité de Génétique des Génomes Bactériens, CNRS URA 2171, Paris, France; 2 Institut Pasteur, Unité de Biologie des Bactéries Pathogènes à Gram Positif, CNRS URA 2172, Paris, France; Dartmouth Medical School, United States of America

## Abstract

We have characterized a novel pleiotropic role for CymR, the master regulator of cysteine metabolism. We show here that CymR plays an important role both in stress response and virulence of *Staphylococcus aureus*. Genes involved in detoxification processes, including oxidative stress response and metal ion homeostasis, were differentially expressed in a Δ*cymR* mutant. Deletion of *cymR* resulted in increased sensitivity to hydrogen peroxide-, disulfide-, tellurite- and copper-induced stresses. Estimation of metabolite pools suggests that this heightened sensitivity could be the result of profound metabolic changes in the Δ*cymR* mutant, with an increase in the intracellular cysteine pool and hydrogen sulfide formation. Since resistance to oxidative stress within the host organism is important for pathogen survival, we investigated the role of CymR during the infectious process. Our results indicate that the deletion of *cymR* promotes survival of *S. aureus* inside macrophages, whereas virulence of the Δ*cymR* mutant is highly impaired in mice. These data indicate that CymR plays a major role in virulence and adaptation of *S. aureus* for survival within the host.

## Author Summary

*Staphylococcus aureus* is a very harmful human pathogen that is a major cause of nosocomial infections. Humans have developed sophisticated defense strategies against invading bacteria, including the innate immune response, with the generation of an oxidative burst inside phagocytic cells. Staphylococcal infections are extremely difficult to eradicate due to the remarkable capacity of these bacteria to adapt to different environmental conditions both inside and outside the host organism. Sulfur metabolism is essential for all living organisms and is tightly controlled by regulatory proteins. In this paper, we revealed an important role for CymR, a major regulator of sulfur metabolism, in adaptation of *S. aureus* to the host environment. Inactivation of the gene encoding this regulator in *S. aureus* leads to a mutant bacterium with increased vulnerability to stress conditions including oxidative stress encountered inside the host. More importantly, the deletion of the *cymR* gene strongly affected the interaction of *S. aureus* with its host, leading to impaired virulence in mice. Our results place CymR among the potential targets for attenuation of *S. aureus* infections.

## Introduction

Cysteine, an important sulfur-containing amino acid, plays a major role in cellular physiology. Cysteine residues are required for the biogenesis of [Fe-S] clusters, are found in the catalytic sites of several enzymes and assist in protein folding and assembly through disulfide bond formation [1], [2]. In several pathogenic bacteria, links between bacterial virulence and cysteine metabolism have been described. In toxinogenic clostridia and *Bordetella pertussis*, toxin synthesis is repressed in the presence of cysteine [3]–[5]. Sulfur metabolism genes are also induced upon interaction of *Neisseria meningitidis* and *Mycobacterium tuberculosis* with human cells [6], [7] and decreased virulence of mutants inactivated in various steps of sulfur metabolism has been reported in several microorganisms [6], [8], [9].

Cysteine-containing molecules such as thioredoxin and glutathione play an important role in protecting cells against oxidative stress [10], [11]. In Gram-positive bacteria, mycothiol, coenzyme A and bacillithiol are thought to function as antioxidant thiols [12]–[14]. Several studies have shown that cysteine itself plays a role in bacterial sensitivity to oxidative stress [15]–[21]. More generally, recent data report the existence of links between cysteine metabolism and the response to various stressors such as hydrogen peroxide, superoxide, diamide, nitric oxide, thiol-reactive electrophiles and metal ions [18], [20], [22]–[24].

Due to the reactivity of the SH group of cysteine and to its toxicity, cysteine metabolism is tightly controlled in bacteria. The CymR repressor, belonging to the poorly characterized Rrf2 family of regulators, has recently been identified as the master regulator of cysteine metabolism in *Bacillus subtilis* and *Staphylococcus aureus* [15], [25]. CymR forms a regulatory complex with the key cysteine biosynthesis enzyme, CysK (*O*-acetyl-serine (OAS) thiol-lyase), to repress genes involved in cysteine formation pathways [26]. We have recently compared the expression profiles of the *S. aureus* Δ*cymR* mutant and the parental SH1000 strain grown in the presence of cystine to characterize global changes in gene expression. The presence of cystine corresponds to conditions where the CymR repressor is active and binds to its direct targets [25], [26]. This transcriptome analysis identified sulfur metabolism genes including direct CymR targets and cell envelope associated genes as differentially expressed in the Δ*cymR* mutant. Moreover, we have shown the involvement of the *S. aureus* CymR regulator in utilization of sulfur sources of human origin and its requirement for efficient biofilm formation [25]. This suggested a potential role for this metabolic regulator in adaptation and survival within the host.

*S. aureus* is an important human opportunistic pathogen responsible for a broad spectrum of diseases ranging from food poisoning and minor skin lesions to life-threatening postsurgical infections in humans [27]. This bacterium is a major cause of nosocomial infections of increasing importance due to the spread of antibiotic resistance, particularly methicillin-resistant strains [28]. Oxidative stress is one of the challenges *S. aureus* faces during host infection. Following ingestion by phagocytic cells such as neutrophils and macrophages, bacteria are exposed to an oxidative burst [29]. Reactive oxygen species (ROS), such as superoxide anion (O_2_^−^), hydrogen peroxide (H_2_O_2_), and hydroxyl radicals (⋅OH), may also be generated as by-products of endogenous metabolism. Their actions lead to damage of DNA, proteins and lipids [30]. Several other stressors such as diamide, resulting in disulfide stress, and metal ions or metal-containing compounds, including copper and tellurite, can also induce oxidative stress [31]. Staphylococci are highly resistant to potassium tellurite (K_2_TeO_3_), a selective agent often used for their isolation [18], [32]. Copper is an essential trace element that is toxic to cells at high concentrations and its homeostasis is maintained by copper uptake and efflux systems [33]. Detoxification enzymes that allow transformation of ROS mediate oxidative stress resistance. In particular, in *S. aureus*, there are two cytoplasmic manganese superoxide dismutases, SodA and SodM that catalyse superoxide radical dismutation. Hydrogen peroxide resulting from this reaction is then eliminated by the action of catalase (KatA). Oxidative stress response and metal ion homeostasis are tightly controlled via a complex regulatory network involving the PerR, Fur, Zur, and MntR repressors [34]–[37]. The PerR regulator of peroxide response controls the expression of genes encoding antioxidants and iron storage proteins and predominantly protects *S. aureus* cells against H_2_O_2_-induced oxidative stress [34]. Fur, the ferric uptake regulator, represses iron uptake genes and positively controls catalase expression, helping to prevent formation of the toxic hydroxyl radical via the Fenton reaction [35]. In addition, the *sodA* and *sodM* superoxide dismutases genes are directly regulated by SarA, a global virulence regulator [38], [39].

In this study, we show that the pleiotropic CymR repressor plays an important role in the response to various stresses and in virulence of *S. aureus*. Comparative transcriptome analysis showed the significant upregulation of genes involved in detoxification processes, including oxidative stress response and metal ion homeostasis in a Δ*cymR* mutant. Increased sensitivity of the Δ*cymR* mutant to H_2_O_2_, disulfide, tellurite and copper stresses might be explained by profound metabolic changes in this mutant. We observed increased survival of the Δ*cymR* mutant inside macrophages but drastically decreased virulence in mice. This indicates for the first time in *S. aureus* the existence of a direct link between the control of cysteine metabolism and adaptation to the host.

## Results

### Upregulation of genes involved in oxidative stress response and metal ion homeostasis in the Δ*cymR* mutant

We have revisited our previously reported expression profiling of Δ*cymR* mutant and parental SH1000 strains focusing on the role of CymR in the *S. aureus* stress response. A more detailed analysis of these transcriptome data, carried out by hierarchical clustering (see Materials and Methods), not only showed derepression of directly CymR-dependent sulfur metabolic genes in the Δ*cymR* mutant [25], but also revealed increased expression of genes involved in detoxification processes such as oxidative stress response and metal ion homeostasis. These include genes belonging to the PerR regulon such as *ahpFC*, *trxB*, *ftnA*, *dps*, *perR* and *fur* [34] as well as the *sodA* and *sodM* genes encoding superoxide dismutases [39] (Table 1). Notably, the *copAP* operon encoding a copper efflux system [33] is also strongly up-regulated in the Δ*cymR* mutant. Quantitative RT-PCR analysis for selected genes was in accordance with transcriptome data and the ratios obtained were generally greater than those resulting from the expression profiling. In particular, we observed about a ten-fold derepression of the *ahpF* and *copA* genes in the *ΔcymR* mutant (Table 1). In addition, several genes differentially expressed under H_2_O_2_, nitrosative, disulfide or paraquat stress conditions also showed altered expression in the Δ*cymR* mutant as compared to the parental strain (Tables 1 and S1).

**Table 1:** Stress response associated genes differentially expressed in the *S. aureus* Δ*cymR* mutant strain compared to SH1000.

Gene name^a^ (synonym)	Function/similarity	Transcriptome analysis^b^		qRT-PCR^c^		Differential expression under stress conditions^d^
		Δ*cymR*/SH1000 expression ratio	*P* value	Δ*cymR*/SH1000 expression ratio		
				TSB+ Cys	TSB	
SA0366 ahpC*	alkyl hydroperoxide reductase, subunit C	5.88	<1.0E-16			Nitrosative, nitrite, H_2_O_2_, diamide, paraquat
SA0365 ahpF*	alkyl hydroperoxide reductase, subunit F	4.80	<1.0E-16	8.52	1.73	Nitrosative, nitrite
SA2481	rhodanese family protein	5.55	<1.0E-16			
SA2345 *copP*	copper-ion-binding protein	3.85	<1.0E-16			H_2_O_2_, nitrite
SA2344 *copA*	cation-transporting ATPase, E1-E2 family	3.54	<1.0E-16	12.48	1.35	H_2_O_2_, nitrite
SA1709 *ftnA*	ferritin	3.00	<1.0E-16			H_2_O_2_, nitrosative, nitrite
SA1941 dps*	general stress protein Dps	2.83	<1.0E-16	4.51	1.44	H_2_O_2_, nitrosative, nitrite
SA1382 sodA*	superoxide dismutase	2.75	<1.0E-16	4.22	1.06	Paraquat, nitrite
SA0128 *sodM*	superoxide dismutase	1.52	1.16E-06	2.29	1.07	Paraquat, nitrite
SA0719 trxB*	thioredoxin reductase	1.70	3.07E-13	3.04	1.27	Nitrite, diamide, paraquat
SA0992 *trxA*	thioredoxin	1.62	3.54E-08	1.85		Nitrite
SA1979*	iron-compound ABC transporter component	1.58	3.92E-05			Nitrosative
SA0758	thioredoxin, putative	1.48	2.52E-06			
SA0755	organic hydroperoxide resistance protein	1.44	1.07E-09			H_2_O_2_
SA0231	flavohemoprotein, putative	1.42	5.29E-07			H_2_O_2_, nitrosative, nitrite
SA1329 fur*	Fur regulator	1.42	0.000036	2.37	1.42	Nitrite
SA1678 perR*	PerR regulator	1.42	0.0012	2.07	1.16	
SA0531 *proP*	osmoprotectant proline transporter	0.47	<1.0E-16			Nitrite

**a.** The SA numbers (N315 strain) for *S. aureus* genes correspond to those of AureoList (http://genolist.pasteur.fr/AureoList/).

“*” indicates genes differentially expressed upon internalization of *S. aureus* in human epithelial cells [58].

**b.** The results obtained are representative of 8 hybridizations from 4 independent cultures in TSB medium with 2 mM cystine. The generated data sets were loaded into the GenoScript Database (http://genoscript.pasteur.fr)[25].

**c.** For qRT-PCR analysis, total RNA was extracted from *S. aureus* strains grown in TSB medium with or without 2 mM cystine. After reverse transcription, specific cDNAs was quantified by qRT-PCR using 16S rRNA gene for normalization.

**d.** Differential expression of corresponding genes/proteins under indicated stress conditions including hydrogen peroxide (H_2_O_2_) [46], [47], diamide [47], paraquat [39], [47], nitrite [68] and nitrosative [69] stresses.

The CymR regulator functions as a transcriptional repressor. Similar levels of derepression in the Δ*cymR* mutant were observed for stress-related genes and for previously identified direct targets of CymR [25]. To determine whether CymR also directly controls stress-related genes, we performed electrophoresis mobility shift assays (EMSAs) with promoter regions of the *copA*, *ahpC*, *sodA*, *ftnA* and *dps* genes using crude extracts of a *S. aureus* Δ*cymR* mutant overexpressing or not *cymR*. We have previously used this approach successfully to demonstrate specific direct interactions of CymR with several promoter regions [25]. No specific DNA-protein complexes for the promoters of stress-related genes were formed under these conditions, indicating that these genes are likely to be controlled indirectly by CymR (data not shown). This is in agreement with the absence of a CymR binding motif in the promoter regions of stress-related genes [25]. CymR also controlled the synthesis of the two oxidative stress regulators, Fur and PerR, and several genes derepressed in the Δ*cymR* mutant belong to the PerR regulon (*ahpFC*, *trxB*, *dps*, *ftnA*). However, CymR does not appear to bind directly to the promoter regions of either *fur* or *perR* in EMSAs (data not shown).

To determine the relative roles of CymR, PerR and Fur on the expression of stress-related genes, we carried out quantitative RT-PCR analysis of gene expression in various mutants inactivated for the *cymR*, *perR* and/or *fur* genes. While some genes of the PerR regulon, such as *dps* and *ahpF*, are strongly repressed by PerR and negatively affected to a lesser extent by Fur and CymR, we did not observe any synergistic effect of the combined mutations. On the contrary, these experiments revealed an antagonistic effect of the Δ*cymR* and Δ*perR* mutations on the expression of the *dps* and *ahpF* genes (Table S2). These genes, belonging to the PerR regulon, were strongly derepressed in a *perR* mutant as compared to the SH1000 strain. However, in a *perR cymR* mutant, the derepression of these genes was lower, and similar to that observed in the Δ*cymR* mutant suggesting antagonistic effects of these two mutations. Inactivation of CymR had similar effects in different mutant backgrounds on the expression of other genes including *sodA*, *sodM* and *copA*. Thus, the CymR effect on stress response does not appear to be mainly mediated by the known oxidative stress regulators, PerR and Fur, even though these regulatory systems may interfere with each other.

To compare the changes induced by peroxide stress in the Δ*cymR* mutant and the parental SH1000 strain, we analyzed the expression of several stress-related genes after an H_2_O_2_ challenge (Table S2). As expected, we observed a strong induction of *ahpF* and *dps* in strain SH1000. However, the induction of *ahpF* expression by H_2_O_2_ was lost in a Δ*cymR* mutant while the extent of *dps* induction was reduced in this mutant as compared to SH1000. This illustrated that the *cymR* deletion resulted in an altered stress response at the molecular level.

### Increased sensitivity of the Δ*cymR* mutant to disulfide, tellurite, copper and H_2_O_2_ stresses

**Figure 1:**
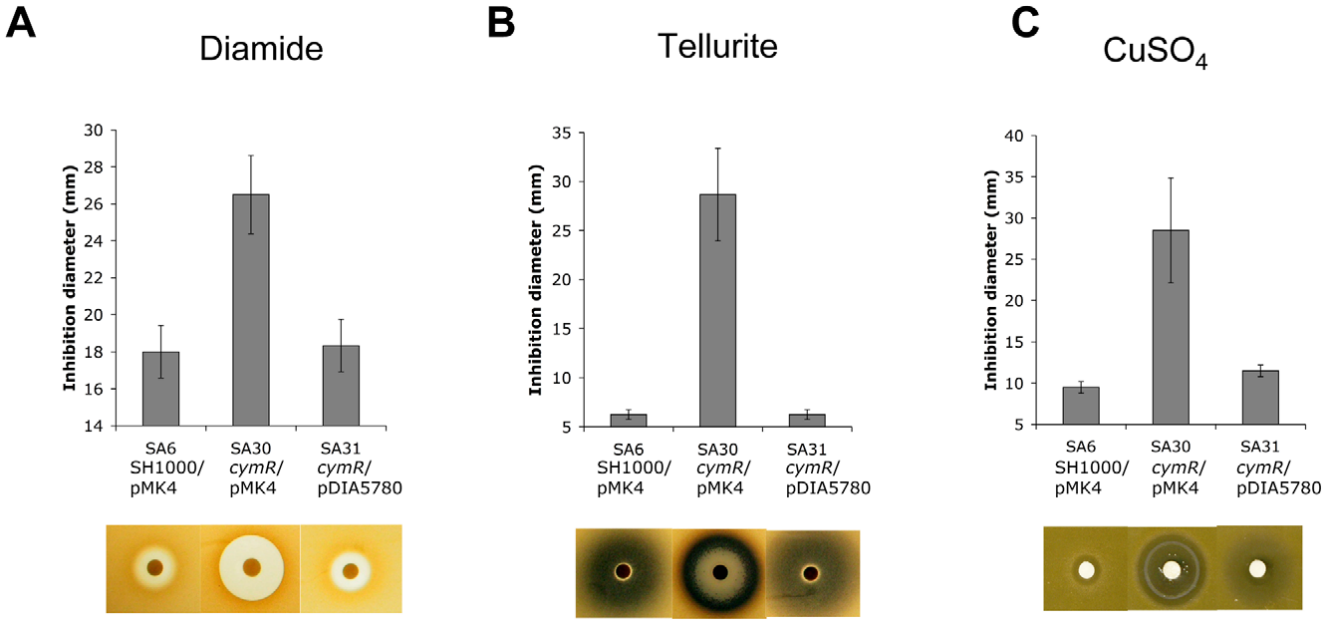
Stress resistance phenotypes of a *S. aureus* Δ*cymR* mutant. Disk diffusion assays were performed with 1 M diamide (**A**), 200 mM tellurite K_2_TeO_3_ (**B**) or 200 mM CuSO_4_ (**C**) in TSB medium. Quantitative analysis of growth inhibition is shown in the upper panels. The lower section shows representative results of stress sensitivity assays. Strains SA6 (SH1000/pMK4), SA30 (Δ*cymR*/pMK4) and SA31 (Δ*cymR*/pDIA5780) were used for complementation experiments. Results correspond to the mean values with standard deviations and are representative of at least three independent experiments.

*S. aureus* can survive a wide range of stresses during its life cycle [29]. As mentioned above, a set of genes involved in stress response was differentially expressed in the Δ*cymR* mutant as compared with the parental strain. The role of CymR in responses to various stress stimuli was tested using either disk diffusion assays or survival analyses. In disk diffusion assays, the Δ*cymR* mutant was significantly more sensitive than SH1000 to 1 M diamide, a specific thiol oxidant that causes disulfide stress (Fig. 1A). The SH1000 strain showed high resistance to 200 mM K_2_TeO_3_ in disk diffusion assays without a detectable growth inhibition area around the 6 mm-disk. By contrast, the Δ*cymR* mutant was extremely sensitive to tellurite stress with a growth inhibition area of 28 mm under the same conditions (Fig. 1B). Bacterial detoxification of tellurite leads to formation of insoluble tellurium (Te°), appearing as black deposits in the growth plates. We also observed increased sensitivity of the Δ*cymR* mutant to copper stress in disk diffusion assays carried out with 200 mM CuSO_4_ (Fig. 1C). Sensitivity of the Δ*cymR* mutant to other metal ions (FeCl_3_, Pb(CH_3_COO)_2_, MnSO_4_, CoCl_2_, ZnSO_4_, HgSO_4_ and NiSO_4_) was similar to that of the parental strain (data not shown). In all cases, increased stress sensitivity of the Δ*cymR* mutant could be complemented by the introduction of plasmid pDIA5780 carrying the *cymR* gene (Fig. 1).

**Figure 2:**
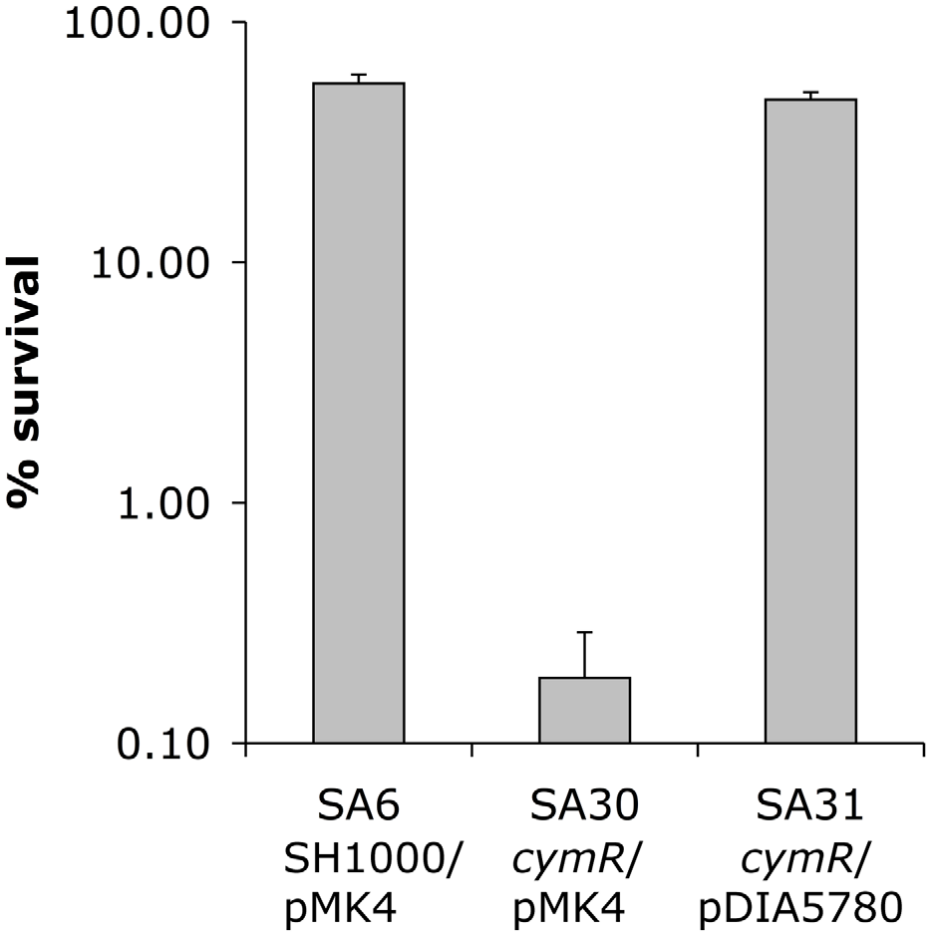
Oxidative stress sensitivity of a *S. aureus* Δ*cymR* mutant. Viability of SA6 (SH1000/pMK4), SA30 (Δ*cymR*/pMK4) and SA31 (Δ*cymR*/pDIA5780) was tested. Exponential-phase cells grown in TSB medium with 2 mM cystine were treated for 1 h with 20 mM H_2_O_2_ and plated on BHI. Results represent the mean values for survival with standard deviations and are representative of at least two independent experiments.

With respect to oxidative stress, no significant differences in sensitivity were observed between the Δ*cymR* mutant and the parental strain in disk diffusion assays in the presence of paraquat (2 M methyl viologen) (data not shown). Viability of the Δ*cymR* mutant and the SH1000 strain grown in TSB medium with cystine was also tested 1 h after addition of 20 mM H_2_O_2_. A 1000-fold reduction in survival was observed for the Δ*cymR* mutant as compared to the parental strain and viability was restored in a Δ*cymR* mutant complemented by pDIA5780 (Fig. 2). We further tested the oxidative stress response in mutants inactivated for CymR, PerR and/or Fur. We observed a 10-fold decreased viability in a Δ*perR ΔcymR* mutant as compared to the Δ*cymR* mutant and extremely low survival capacities for the Δ*cymR* Δ*perR* Δ*fur* mutant as compared to the Δ*perR* Δ*fur* mutant (data not shown). Taken together, our results indicate that CymR plays a major role in staphylococcal stress response, independently of other known regulators.

Increased oxidative stress sensitivity of the Δ*cymR* mutant could be due to elevated intracellular cysteine pools, driving production of hydroxyl radicals via the Fenton reaction and leading to cellular damage [20]. We tested the effect of cystine on stress sensitivity in a Δ*cymR* background. We observed decreased sensitivity to tellurite and copper stress of the Δ*cymR* mutant in the presence of cystine in several genetic backgrounds including *perR* and/or *fur* mutants (Fig. S1 and S2). By contrast, the Δ*cymR* mutant showed increased sensitivity to both diamide and H_2_O_2_ stress in the presence of cystine (data not shown). Similarly, the decrease in H_2_O_2_ and diamide stress resistance in the Δ*cymR* mutant due to *perR* inactivation was more pronounced in the presence of cystine (data not shown). In agreement with altered oxidative stress response, the presence of cystine affected the expression of genes associated with stress response in the Δ*cymR* mutant as shown by quantitative RT-PCR analysis (Table 1). These genes were differentially expressed in a Δ*cymR* mutant only in the presence of cystine.

### Metabolic changes of the Δ*cymR* mutant in the presence of cystine

**Figure 3:**
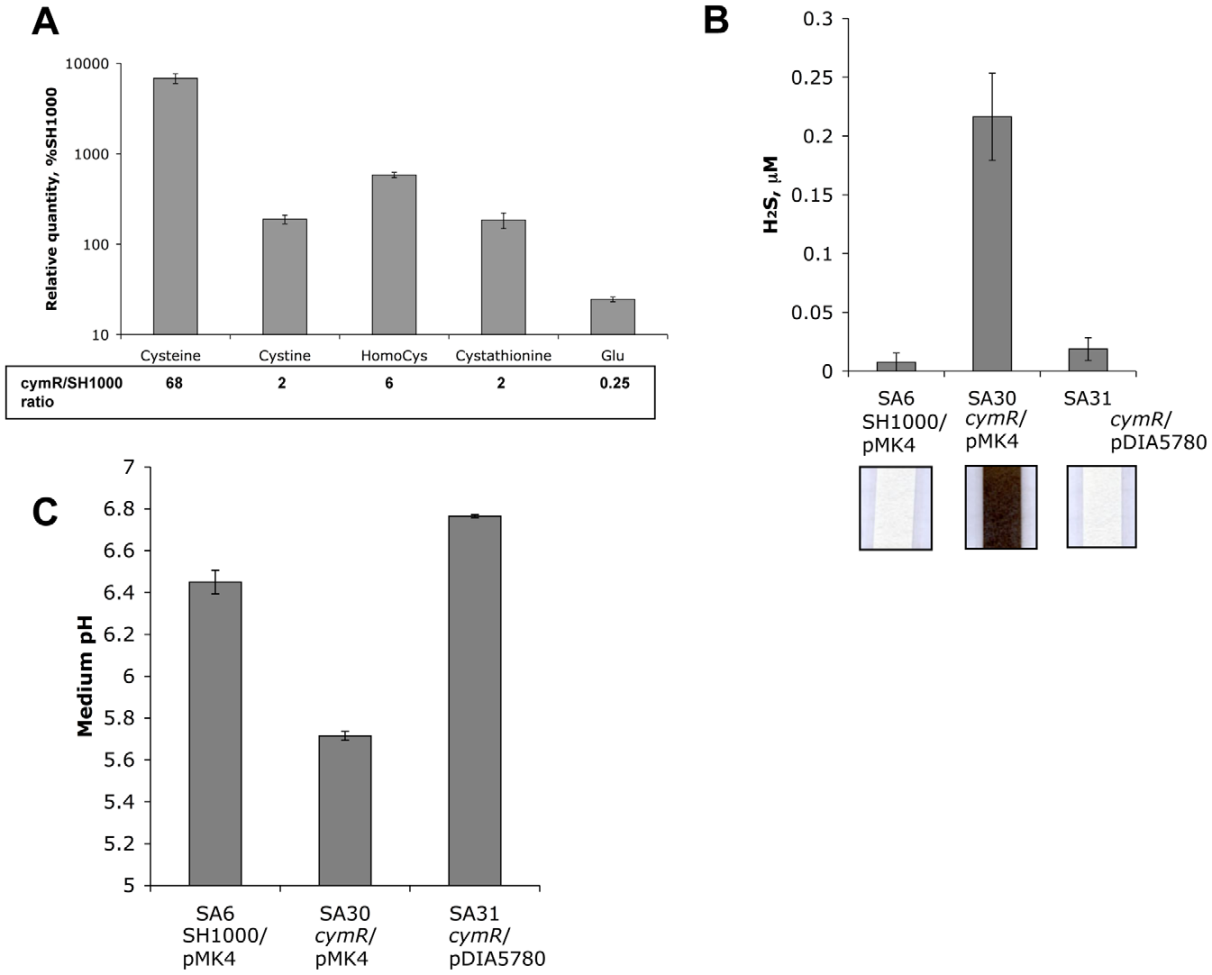
Metabolic changes in the Δ*cymR* mutant after growth in the presence of cystine. The strains were grown in TSB medium with 2 mM cystine. **A**. Intracellular metabolite concentrations were estimated by HPLC for the Δ*cymR* mutant (SA17) and the parental SH1000 strain. The Δ*cymR*/SH1000 ratio is indicated. The complete data on metabolite concentrations are given in Table S3. **B**. H_2_S production measurement was performed using the quantitative methylene blue method and a Na_2_S standard curve. Representative results of lead-acetate paper assays are shown in the lower section. Strains SA6 (SH1000/pMK4), SA30 (Δ*cymR*/pMK4) and SA31 (Δ*cymR*/pDIA5780) were used. **C**. The pH of the medium was measured after an overnight culture (16 h) at 37°C. Strains SA6 (SH1000/pMK4), SA30 (Δ*cymR*/pMK4) and SA31 (Δ*cymR*/pDIA5780) were used. Results correspond to the mean values with standard deviations and are representative of at least two independent experiments.

The altered stress response linked to *cymR* inactivation can be explained by an imbalance in thiol redox status. The derepression of genes involved in cystine uptake and cysteine biosynthesis from sulfide and homocysteine may result in cysteine accumulation in the Δ*cymR* mutant. Analysis of the intracellular pools of several metabolites using HPLC revealed a strong up to 68-fold increase of the intracellular cysteine concentration in the Δ*cymR* mutant in comparison with the parental strain during growth in TSB medium with cystine (Fig. 3A and Table S3). We also observed a 2-fold increase in cystine and cystathionine content and a 6-fold increase in homocysteine content. This analysis also revealed a 36-fold increase in the cysteine to cystine ratio in the Δ*cymR* mutant as compared to the SH1000 strain, reflecting the imbalance in thiol redox status of the cell in the absence of CymR. The estimated glutamate concentration decreased 4-fold while the concentration of other amino acids was unchanged in the Δ*cymR* mutant as compared to the SH1000 strain. As cysteine is probably toxic for the cell at high concentrations, it may then be rapidly transformed into hydrogen sulfide, pyruvate and ammonia by cysteine desulfhydrases. The MccB, MetC and CysK enzymes have cysteine desulfhydrase activities in *B. subtilis* [40] and orthologous proteins are present in *S. aureus*. We then compared production of hydrogen sulfide, the main product of cysteine catabolism, in the Δ*cymR* mutant and SH1000 strains grown in the presence of cystine. An important increase in H_2_S production was observed in the Δ*cymR* mutant in a qualitative lead-acetate-paper assay and a 40-fold increase was further confirmed by an H_2_S quantification assay (Fig. 3B). The introduction of a plasmid carrying the intact *cymR* gene into the Δ*cymR* mutant led to a level of H_2_S production similar to that observed in the parental SH1000 strain (Fig. 3B). In the absence of cystine, H_2_S production was undetectable in the Δ*cymR* mutant and SH1000 strains (data not shown). We also measured the pH of the TSB medium after 16 h culture in the presence of cystine. A significant acidification of the medium was observed with the Δ*cymR* mutant as compared with the parental strain (Fig. 3C). This may be associated with pyruvate production from cysteine and/or with a decreased capacity to catabolize organic acids. These changes in the pH of the growth medium of the Δ*cymR* mutant could be reversed by the introduction of a plasmid carrying the *cymR* gene.

### The Δ*cymR* mutation favors *S. aureus* survival within macrophages

**Figure 4:**
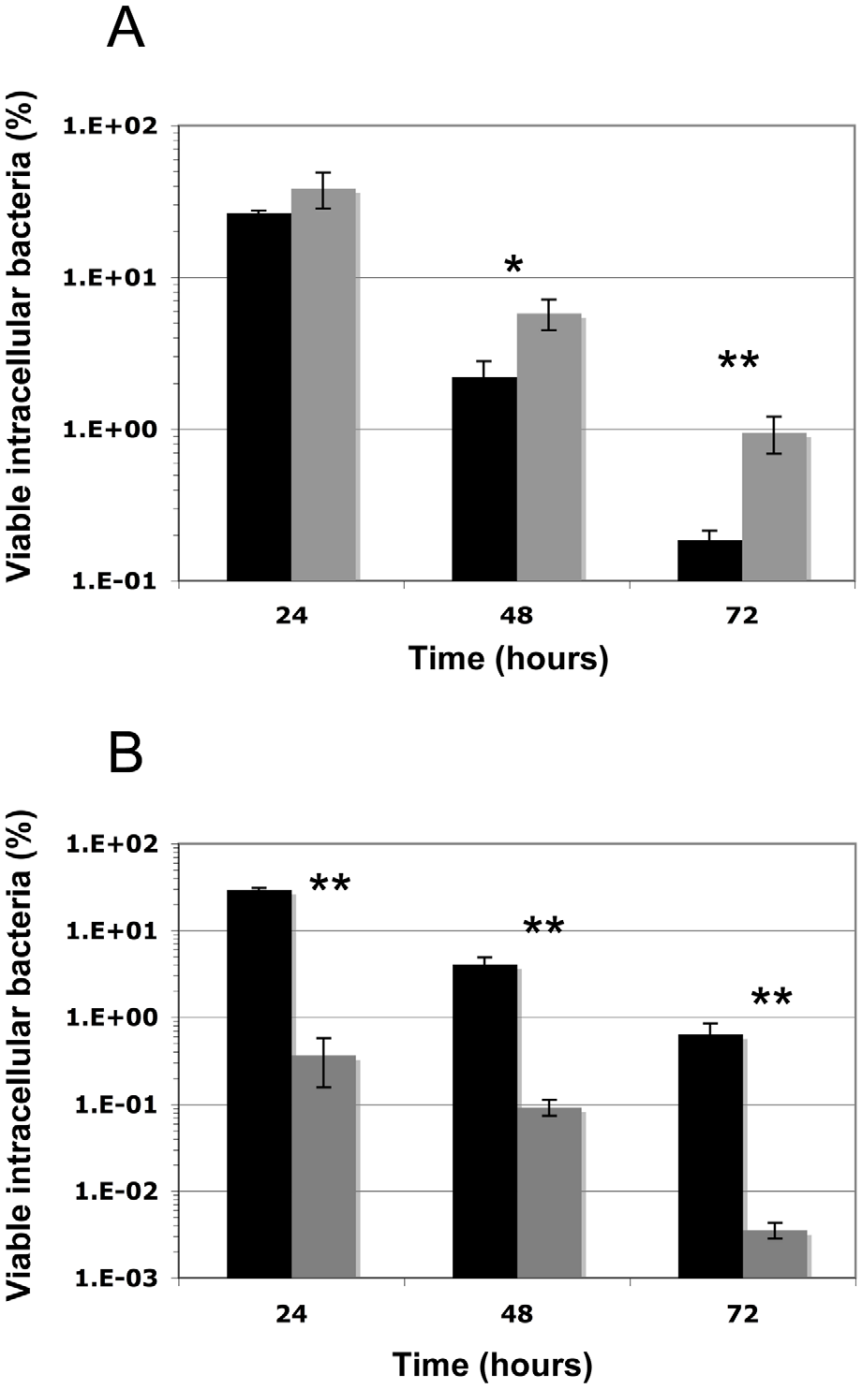
Intracellular survival of *S. aureus* SH1000, the Δ*cymR* and *sodAsodM* mutants in RAW 264.7 macrophages. Macrophages were infected as described (See Materials and Methods) with the SH1000 parental strain (black) or the Δ*cymR* mutant (panel A) or the *sodAsodM* mutant (panel B) (grey). Viable intracellular bacteria at 24, 48, and 72 h were counted and expressed as a percentage of internalized bacteria. One representative experiment (out of 3) is shown, performed in triplicate (means±SD). *: P<0.05; **: P<0.01.

Since the CymR regulator was shown to be involved in stress adaptation, we tested its role in the *S. aureus* survival inside macrophages. Professional phagocytes are the first line of defense encountered by pathogens during the infection process. Since it has been shown that *S. aureus* is particularly efficient in persisting within professional phagocytes [41], [42], the survival of the parental SH1000 strain and the Δ*cymR* derivative inside RAW 264.7 murine macrophages was investigated over a 3-day period (Fig. 4A). We also examined the survival of a *sodAsodM* mutant as a positive control of macrophage stress generation. As shown in Fig. 4B, clearance of the *sodAsodM* mutant was much faster than that of the wild type strain, directly correlating its increased stress sensitivity with lowered survival within RAW 264.7 murine macrophages. We measured the internalization rates of the parental and Δ*cymR* mutant strains, which were identical (about 90% of entry for a multiplicity of infection (m.o.i)  = 5). Viable bacterial counts inside macrophages over time allowed us to demonstrate that the Δ*cymR* mutant is more resistant to macrophage stress than the parental strain (Fig. 4A).

### The Δ*cymR* mutation has a drastic effect on *S. aureus* virulence

**Figure 5:**
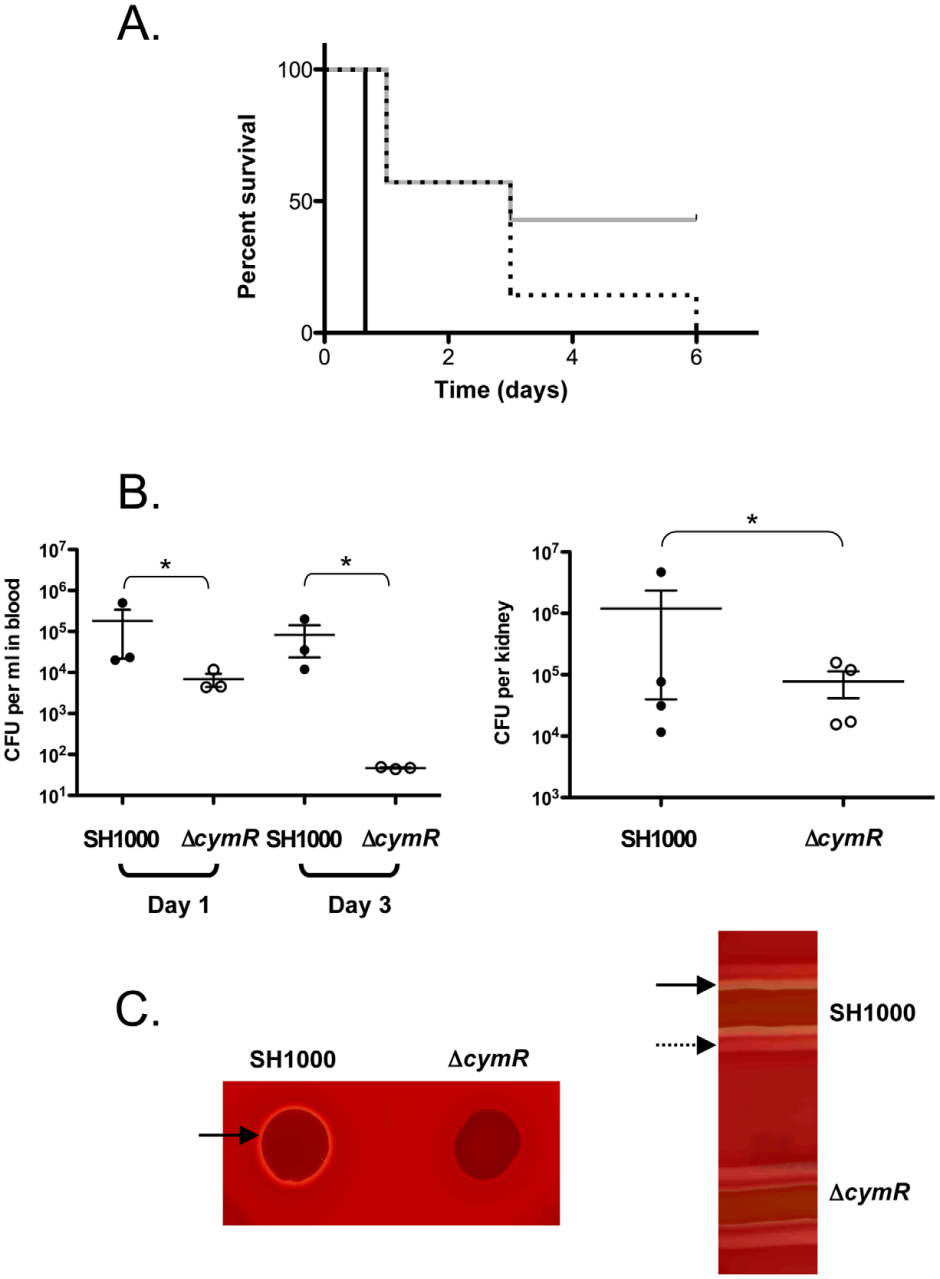
Role of CymR during *S. aureus* infection. **A**. Survival of BALB/c mice following intraperitoneal challenge with 3.10^8^ CFU of the SH1000 parental strain (black), the Δ*cymR* derivative (grey), and the *sodAsodM* mutant (dotted lines). Comparison of survival curves was performed using the log-rank test (P<0.005). Results are representative of at least two independent experiments. **B**. Bacterial counts of the SH1000 strain and the Δ*cymR* mutant in blood (left panel) and kidneys (right panel). BALB/c mice were infected by the i.p. route with 5.10^7^ CFU of each strain. Bacteraemia was measured 1 and 3 days post-infection, and the bacterial load in kidneys was determined 7 days post-infection. Bars represent mean CFU. *, P>0.001 (Student's t test). **C**. Hemolytic activity assays. Overnight cultures of the SH1000 wild type strain and the Δ*cymR* derivatives were spotted (20 µl) on horse blood agar plates or streaked on sheep agar plates. Full and dotted arrows indicate δ- and β-hemolysins, respectively.

Professional phagocytes are part of the host anti-microbial defense and the Δ*cymR* mutation seems to favor intracellular survival of *S. aureus*. We therefore tested whether this selective advantage could have an effect on global *S. aureus* virulence, using a murine intraperitoneal infection model with BALB/c mice since this lineage has been shown to be susceptible to *S. aureus* infection [43]. We infected mice intraperitoneally with 3.10^8^ colony-forming units (CFU) of either the SH1000 strain or the Δ*cymR* mutant. As shown in Fig. 5A, while mice infected by the parental strain were all dead (7 mice/7) 18 h after inoculation (black curve), those infected with the Δ*cymR* mutant displayed a significant extension of time-to-death and 3 mice (out of 7) were still alive 6 days post-infection (grey curve). As a control, we tested a *sodA sodM* mutant previously described as impaired in its capacity to develop abscesses in a mouse subcutaneous infection model [39]. We observed that decreased mouse mortality linked to the bacterial *sodA sodM* inactivation was equivalent to that caused by *cymR* inactivation (Fig. 5A, dotted lines). In order to follow bacterial dissemination within the animal, we also infected mice with a sub-lethal dose (5.10^7^ CFU) of either the SH1000 strain or the Δ*cymR* mutant. We followed bacteraemia at 1 and 3 days post-infection and quantified the renal load 7 days post-infection. The bacterial load drastically decreased in the *cymR* mutant (Fig. 5B). In the blood, one -day post-infection, there was at least 1-log-unit decrease for the Δ*cymR* mutant compared to the wild type strain (Fig. 5B, left panel). The difference between the two strains increased 3 days post-infection with more than 3-log-unit less bacteria in the blood in a Δ*cymR* background. The bacterial load in the kidneys also showed a colonization defect of the Δ*cymR* mutant since 7 days post-infection there was more than 1-log-unit less CFU with the Δ*cymR* strain than with SH1000 (Fig. 5B, right panel). Thus, although the Δ*cymR* mutation appears to be beneficial with regard to survival within professional phagocytes, it largely decreases global *S. aureus* virulence.

In order to determine whether the production of major virulence factors is affected in the Δ*cymR* mutant, we performed hemolytic activity assays on blood agar plates. These experiments clearly show that the Δ*cymR* mutant is impaired in its capacity to produce δ-hemolysin as compared to the parental SH1000 strain (Fig. 5C, left panel). On sheep blood agar plates, we can distinguish between δ- and β-hemolysin [44]. As shown on Fig. 5C (right panel) we have confirmed that the δ-hemolysin production was strongly reduced in the *cymR* mutant strain with respect to the parental strain, whereas β-hemolytic activity was not affected by the mutation. α-hemolytic activity was not tested since it is inhibited by β-hemolysin [45]. This significantly altered δ-hemolysin production likely contributes to the virulence defect observed in the absence of CymR.

## Discussion

In *S. aureus*, diamide and H_2_O_2_-induced stresses result in induction of several direct CymR target genes including *mccAB*, *cysM*, *tcyABC* and *metNPQ*, indicating an increased requirement for cysteine under these conditions [46], [47]. Conversely, here we show an upregulation of part of the peroxide stress PerR regulon, of superoxide stress (*sodA* and *sodM*) and copper efflux system (*copAP*) genes together with other stress-related genes in a *S. aureus* mutant lacking the master regulator of cysteine metabolism, CymR. However, the effect of CymR on these genes appears to be indirect. We investigated possible connections between CymR and the PerR and Fur regulators of oxidative stress response. CymR appears to affect stress response independently of these regulators, since the effect of *cymR* inactivation on stress sensitivity and gene expression is still observed in *perR* and/or *fur* mutant backgrounds.

**Figure 6:**
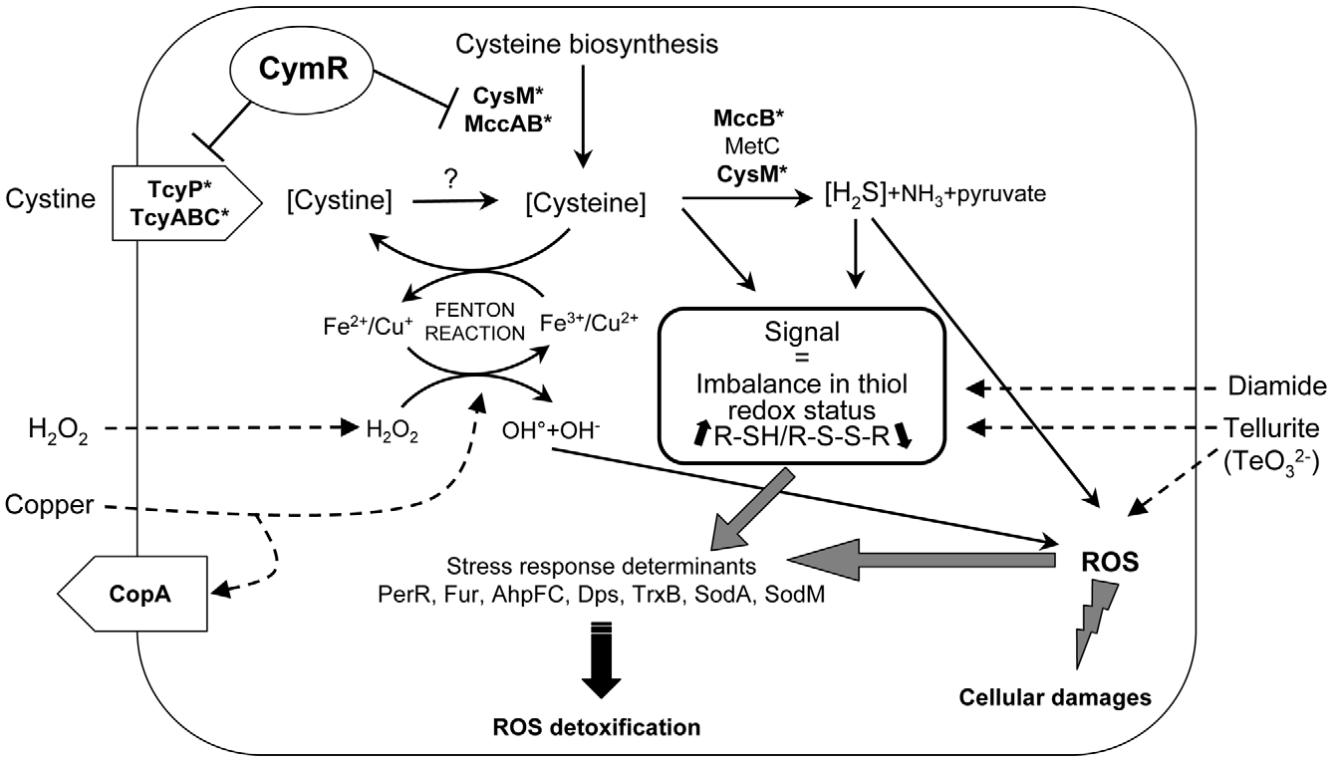
Proposed model for the role of CymR in the stress response in *S. aureus*. In the absence of CymR, the derepression of genes involved in pathways leading to cystine/cysteine uptake (e.g. *tcyP*, *tcyABC* encoding cystine transporters) and biosynthesis (e.g. *cysM* encoding OAS thiol-lyase and *mccAB* encoding homocysteine to cysteine conversion enzymes) leads to increased intracellular cysteine levels. High amounts of cysteine promote oxidative DNA damage by driving the Fenton reaction. Iron and copper are both capable of catalyzing the formation of hydroxyl radicals from H_2_O_2_. Cysteine may be catabolized into hydrogen sulfide (H_2_S), pyruvate and ammonia (NH_3_) by cysteine desulfhydrases (MccB, MetC, CysM). High H_2_S levels may also induce oxidative stress by the formation of reactive oxygen species (ROS). The altered stress response may be explained by an imbalance in thiol redox status induced by *cymR* inactivation. Different stress response systems including PerR regulon members (AhpFC, Dps, TrxB, PerR and Fur), superoxide dismutases (SodA, SodM) and a copper efflux system (CopA) may recognize a common stress signal in the Δ*cymR* mutant. This signal may either be an increase in intracellular cysteine pools or changes in levels of other cysteine-related compounds. Tellurite, copper, H_2_O_2_ and diamide can cause imbalance in the thiol status of the cytoplasm and oxidative stress. A question mark indicates a step, which remains to be characterized. Asterisks indicate directly controlled CymR target genes.

We propose that these different stress response systems may recognize a common stress signal that is present in the Δ*cymR* mutant. This signal could be related to thiol-redox homeostasis imbalance and to increases in intracellular cysteine pools or changes in other cysteine-related compound content including H_2_S (Fig. 6). Cysteine is one of the major cellular thiols in *S. aureus*. Metabolite content estimation revealed a 36-fold increase in the cysteine to cystine ratio in the Δ*cymR* mutant reflecting the imbalance in thiol redox status in the absence of CymR. It is worth noting that the simultaneous induction of the PerR and CymR regulons as well as metal-ion efflux systems, including CopA, by thiol-reactive electrophiles leading to imbalance of thiol-redox homeostasis has been reported in *B. subtilis* [24]. In agreement with this metabolic hypothesis, the addition of cystine to the culture medium affected stress-related phenotypes of the Δ*cymR* mutant. Recent studies suggested the existence of links between cysteine and/or cysteine-containing molecules and oxidative stress defense in several bacterial systems with positive or negative effects of this amino acid. Cysteine protects *Lactobacillus reuteri* from H_2_O_2_ stress while cysteine or thiol-derived compounds such as glutathione are important for defense against damages [10], [19]. By contrast, in *E. coli*, a 8-fold increase in intracellular cysteine concentrations promotes oxidative DNA damages by driving the Fenton reaction due to the efficient reduction of Fe^3+^ by cysteine [20]. We observed a strong 68-fold increase in the intracellular cysteine pool in the Δ*cymR* mutant grown in the presence of cystine, leading to a 1000-fold increase in sensitivity to H_2_O_2_ stress. However, the addition of extracellular or cell-penetrating iron and copper chelators (dipyridyl, desferal, neocuproine and ferrozine) had no positive effect on viability of the Δ*cymR* mutant after an H_2_O_2_ challenge (data not shown). This suggests more complex mechanisms of altered stress response in addition to the Fenton reaction-mediated process, as recently proposed for other microorganisms [48], [49]. High cysteine levels are correlated with the production of H_2_S by cysteine desulfhydrases (MccB, MetC, and CysM) (Fig. 6). H_2_S increases the formation of H_2_O_2_ and other ROS in several organisms and inhibits human superoxide dismutase activity and *S. aureus* catalase activity in acid medium [50]–[52]. This could also contribute to the oxidative stress sensitivity of the Δ*cymR* mutant.

The *S. aureus* Δ*cymR* mutant exhibited increased susceptibility to disulfide, copper, tellurite, and H_2_O_2_-induced oxidative stresses. Diamide, tellurite and copper can each cause both oxidative stress as well as an imbalance in the thiol redox status of the cytoplasm (Fig. 6). A recent proteomic study that analyzed the diverse *S. aureus* responses to H_2_O_2_, diamide and paraquat [47] indicates a close relationship between disulfide and H_2_O_2_ stress responses, in agreement with the similar behavior of the Δ*cymR* mutant toward these compounds. Tellurite (TeO_3_^2−^) is toxic for most forms of life, even at very low concentrations. The genetic and biochemical basis underlying bacterial tellurite toxicity is still poorly understood [32]. However, several tellurite resistance determinants have been identified, mainly in *E. coli*, suggesting mechanisms involving cysteine metabolism and cellular oxidative stress due to its strong oxidizing ability. Cysteine synthases from various bacteria and molecules containing cysteine including glutathione are involved in tellurite resistance via reductive detoxification of this compound [32]. In *S. aureus*, the *cysM* mutant defective in cysteine synthase is more sensitive to tellurite, probably due to cysteine depletion [18]. Inactivation of *cymR* also leads to extreme sensitivity to tellurite, even greater than that of the *cysM* mutant. However, the addition of cystine to the culture medium resulted in a drastic decrease in tellurite toxicity in both the *cymR* and *cysM* mutants (Fig. S1 and data not shown). The accumulation of cysteine and/or H_2_S (Fig. 3) under these conditions could promote tellurite detoxification leading to the formation of nontoxic tellurium. As observed with tellurite, a Δ*cymR* mutant is more sensitive to copper stress than the parental SH1000 strain, and this effect is more pronounced in the absence of cystine (Fig. S1). The *copA* and *copP* genes encoding a copper efflux system involved in maintaining copper homeostasis in *S. aureus* [33] are strongly upregulated in the Δ*cymR* mutant in the presence of cystine (Table 1). Further studies will be required to characterize the molecular mechanisms linking CymR to tellurite and copper sensitivity.

The intracellular cysteine level is kept within a narrow range to address both the cysteine supply for protein synthesis and the production of other essential molecules and the necessity of maintaining cysteine levels below the toxicity threshold. Elevated cysteine or H_2_S levels must also be avoided as they may lead to cysteine autooxidation, the production of ROS and protein thiol oxidation [53], [54]. The CymR regulator in *S. aureus* plays an essential role in maintaining intracellular cysteine levels. However, H_2_S together with cysteine may be a signal recognized by several oxidative stress defense systems in *S. aureus*. During infection, this pathogen must cope with host phagocytic attack, accompanied by the release of a number of ROS including superoxide anion, hydrogen peroxide, hydroxyl radical, peroxynitrite and hypochlorous acid [29], [55]. In this study, we showed that the Δ*cymR* mutant has an increased long-term survival rate within macrophages. This result could be related to increased transcription in the Δ*cymR* mutant of a number of genes known to be differentially expressed under several host-related stress conditions, including H_2_O_2_, nitrite and nitrosative stresses (Tables 1 and S1). The differences observed *in vitro* after a H_2_O_2_ challenge and *in vivo* in macrophages may be explained by variations in the level of H_2_O_2_ formed as well as a multitude of reactive species produced inside macrophages. Despite the fact that *cymR* inactivation promotes survival of *S. aureus* inside the macrophages, virulence of the Δ*cymR* mutant in mice is drastically impaired as previously observed for an *S. aureus* strain lacking catalase and beta-toxin [56]. During the infectious process, the CymR regulator influences different virulence pathways. Indeed, we have shown that mice infected with a lethal dose of the SH1000 strain died very rapidly (less than 18 hours post-inoculation), suggesting that toxemia is responsible. Accordingly, we observed that the Δ*cymR* mutant has impaired hemolytic activity. Reduced hemolysin production may be responsible at least in part for the virulence defect observed in the absence of CymR. In addition, bacteraemia and the bacterial load in kidneys following infection with a sub-lethal dose were significantly decreased in the absence of CymR.

Bacterial metabolism has been linked to virulence of Staphylococci by several studies [57]. Some CymR regulon cysteine metabolic genes (*mccA*, *cysM* and *tcyAB*) were shown to be differentially expressed upon internalization of *S. aureus* in human epithelial cells [58]. In the Δ*cymR* mutant, we also observed differential expression of genes known to be affected upon internalization in human cells (Table S4).

Our results suggest that the link between cysteine metabolism control by CymR, stress response and virulence is likely indirect and may be integrated into the general concept that alterations of the bacterial metabolic status create metabolic signals that may be “sensed” by the regulatory network controlling virulence determinants, as proposed by Somerville and Proctor [57]. One hypothesis may be that the alteration in redox cell status and metal ion homeostasis modulates the activity of virulence and stress-response regulators, including SarA, SarZ and PerR. Indeed, recent results have shown that the central virulence regulator, SarA, is responsive to redox and pH [59] and that SarZ is a redox active regulator [60], [61]. Thus, *cymR* inactivation may affect redox-mediated virulence control in *S. aureus* at several levels of the regulatory network. Indeed, a number of genes differentially expressed in strains deficient for virulence regulators (such as SarA, AgrA, ArlSR, SaeSR, Rot and MgrA) showed altered expression in the Δ*cymR* mutant in comparison with the parental SH1000 strain (Table S4).

The role of CymR in virulence is most likely multifactorial since, as we show here, it controls several steps in the infectious process, including dissemination within the host and colonization of different organs. The Δ*cymR* mutant is also affected in biofilm formation and in synthesis of exotoxins (hemolysins) and cell envelope components, functions that could be important for host colonization [25]. Our data bring important insights into understanding the interactions between sulfur metabolism and virulence of this major pathogen and suggest interesting possibilities for metabolic strategies to attenuate *S. aureus* infection. Proteins involved in controlling cysteine metabolism may therefore represent potential targets for antibacterial compounds aimed at treating staphylococcal infections.

## Materials and Methods

### Ethics statement

All the animal experiments described in the present study were conducted at the Institut Pasteur according to the European Union guidelines for the handling of laboratory animals (http://ec.europa.eu/environment/chemicals/lab_animals/home_en.htm) and were approved by the Institut Pasteur animal care and use committee.

### Bacterial strain construction and growth conditions

Bacterial strains used in this study are listed in Table 2. *S. aureus* was grown in brain heart infusion (BHI) (Oxoid) or tryptic soy broth/agar (TSB/TSA) (Difco) [25]. Antibiotics were added at the following concentrations: chloramphenicol, 5 µg ml^−1^; erythromycin, 1 or 5 µg ml^−1^, tetracycline, 5 µg ml^−1^ and kanamycin, 50 µg ml^−1^. *S. aureus* was transformed by electroporation [62]. The chromosomal *perR*, *fur*, *sodA* and *sodM* mutations [34], [35], [39](Table 2) were introduced into the SH1000 strain or ΔcymR mutant by Φ11 phage transduction [63].

**Table 2:** Strains and plasmids used in this study.

Strain or plasmid	Genotype or description	Reference or source
**Strain**		
SH1000	Functional *rsbU+* derivative of 8325-4 wild-type strain	[70]
S897	8325-4 *perR*::*kan* a	[34]
S906	8325-4 *fur*::*tet*	[35]
S1799	SH1000 *sodA*::Tn917(*ery*)	[39]
S739	SH1000 *sodM*::*tet*	[39]
SA17	SH1000 Δ*cymR*	[25]
SA6	SH1000/pMK4	[25]
SA30	SH1000 Δ*cymR*/pMK4	[25]
SA31	SH1000 Δ*cymR*/pDIA5780	[25]
SA37	SH1000 *perR*::*kan*	This study
SA39	SH1000 Δ*cymR perR*::*kan*	This study
SA41	SH1000 *fur*::*tet*	This study
SA42	SH1000 Δ*cymR fur*::*tet*	This study
SA53	SH1000 *perR*::*kan fur*::*tet*	This study
SA61	SH1000 Δ*cymR perR*::*kan fur*::*tet*	This study
SA63	SH1000 *sodA*::*ery sodM*::*tet*	This study
**Plasmids**		
pMK4	*E. coli – S. aureus cat^r^* shuttle vector	[71]
pDIA5780	pMK4 derivative carrying the *cymR* gene for complementation	[25]

*^a^kan*, *ery*, *tet* and *cat* genes encode proteins leading to kanamycin, erythromycin, tetracycline and chloramphenicol resistance.

### Stress response analysis

Disk diffusion assays were performed as follows: 5 ml of TSB or BHI top agar (0.7%, wt/vol) was seeded with 100 µl of an exponential-phase *S. aureus* culture in TSB or BHI medium (OD_600_ = 0.2) and used as an overlay on a TSA or BHI agar plates. When indicated 2 mM cystine was added to the culture medium and to the agar plates. Sterile 6 mm disks were placed on top of the overlay, and 10 µl of either 1 M diamide, 200 mM K_2_TeO_3_, 200 mM CuSO_4_, 10 M H_2_O_2_ or 2 M paraquat (methyl viologen) (Sigma) was added to the disk. Diameters of growth inhibition zones were measured after 24 h of incubation at 37°C. Hydrogen peroxide resistance assays were carried out as previously described with some modifications [18]. Cells were grown in TSB medium with or without 2 mM cystine. At exponential phase (OD_600_ = 0.2), H_2_O_2_ was added to a final concentration of 20 mM in TSB medium. After 1 h of incubation, cells were serially diluted in BHI medium and viability was assessed by overnight growth on BHI agar.

### Hierarchical clustering analysis

Previously obtained transcriptome data [25] were analyzed using hierarchical clustering as the less *a priori*-based method for transcriptome data exploitation. Uncentered Pearson correlation was used for distance calculation, and the average-linkage clustering was performed on logarithmically transformed data for gene expression ratio in SH1000 versus Δ*cymR* mutant. We used the Michael Eisen Cluster software program, followed by tree diagram visualization with TreeView [64]. This analysis revealed several specific clusters including the group of genes upregulated in the Δ*cymR* mutant and involved in detoxification processes.

### Estimation of metabolite content

Strains were grown in TSB medium with 2 mM cystine to an OD_600_ of 1 (with 1/10 medium-to-flask volume ratio at 160 rpm shaking). H_2_S production was revealed using lead-acetate paper (Macherey-Nagel) which turned black following incubation for up to 3 h at 37°C. H_2_S production was quantified by the modified methylene blue reaction as previously described [65]. Intracellular concentrations of amino acids and other ninhydrin-reactive compounds were estimated using high-pressure liquid chromatography (HPLC) [26], [66]. Briefly, cells were suspended in a sulfosalicylic acid buffer (3% final concentration) and disrupted using a FastPrep apparatus (Bio101). Supernatant samples were analyzed by cation-exchange chromatography, followed by ninhydrin postcolumn derivatization as previously described [66].

### RNA extraction and quantitative real-time PCR

Total RNA was isolated from *S. aureus* strains grown in TSB with or without 2 mM cystine as previously described [15]. For H_2_O_2_ stress induction bacteria were incubated with 20 mM H_2_O_2_ for 10 minutes followed by RNA extraction. Quantitative real-time PCR analysis was performed as previously described [25]. Oligonucleotides used in this study are listed in Table S5.

### Electrophoresis mobility shift assays

DNA fragments containing various promoter regions were amplified by PCR using specific primers and chromosomal DNA of *S. aureus* strain SH1000. PCR products were labeled using [γ^32^P]ATP 5′-end labeled specific primers. Protein-DNA complexes were formed in 10 µl reaction volumes, by incubating labeled DNA fragments with various amounts of crude extracts of the *S. aureus* Δ*cymR* mutant carrying either pDIA5780 (pMK4-*cymR*) or pMK4 as previously described [66].

### Macrophage survival assays

Murine macrophage RAW 264.7 cells were used for bacterial survival assays as previously described [67] with some modifications. Briefly, bacteria were grown in TSB until OD_600_ ∼2. Cultures were washed in PBS and adjusted to the desired inoculum in RPMI 1640 medium (Gibco), and CFU counts were verified by plating serial dilutions on TSA plates. Macrophages grown to confluence were counted and incubated with bacteria (m. o. i. ∼5) in RPMI 1640 at 37°C with 5% CO_2_ for 1 h to allow bacterial phagocytosis. They were then washed once with RPMI and incubated in RPMI-10%Fetal Calf Serum-streptomycin (100 µg ml^−1^)/penicillin (100 U ml^−1^). At the indicated times, infected macrophages were washed once with RPMI and then lysed by incubation in ice-cold water for 15 min. CFU counts were determined by plating serial dilutions on TSA plates.

### Mouse virulence assays

Female inbred BALB/c mice (4 to 5 weeks old) were obtained from Janvier Laboratories (Le Genest-St-Isle, France). *S. aureus* strains (SH1000 and the Δ*cymR* or the *sodA sodM* derivatives) were grown in TSB until OD_600_ ∼2, cells were pelleted and resuspended to the appropriate concentration in sterile PBS. Mice were injected by the intraperitoneal route with ∼3.10^8^ CFU (mortality assays), or 5.10^7^ CFU (sub-lethal dose) in 0.2 ml PBS.

For mortality rate assays, mice were monitored daily for signs of illness and death. At the end of the experiment, surviving mice were humanely sacrificed (CO_2_ asphyxiation). Results were statistically analyzed by the log-rank test using Prism 5.0b software (GraphPad Software, San Diego, CA).

For measuring the bacterial load in blood and kidneys, animals were followed during 7 days post-infection. Blood samples were collected from the retro orbital sinus 1 and 3 days post-infection, immediately mixed with heparin and plated on TSA plates. Seven days post-inoculation, mice were sacrificed (CO_2_ asphyxiation), and the kidneys were removed and homogenized for determination of CFU counts.

### Hemolytic activity assays

Hemolysis was detected on Columbia blood agar plates (BioMérieux). Strains were grown overnight in TSB medium and then either spotted (20 µl) on horse blood agar plates or streaked on sheep blood agar plates. The plates were incubated for 24 hours at 37°C, and specific hemolytic activities (β- and δ-hemolysins) were identified as previously described [44].

## Supporting Information

Figure S1 Cystine effect on stress resistance phenotypes of an *S. aureus* Δ*cymR* mutant. Disk diffusion assays were performed with 200 mM tellurite K_2_TeO_3_ (A) or 200 mM CuSO_4_ (B) in TSB medium with (white) and without (grey) 2 mM cystine. Strains SA6 (SH1000/pMK4), SA30 (*cymR*/pMK4) and SA31 (*cymR*/pDIA5780) were used for complementation experiments. Results represent the mean values with standard deviations and are representative of at least three independent experiments.
(0.62 MB TIF)

Figure S2 Cystine effect on stress resistance phenotypes of *S. aureus* mutant strains. Disk diffusion assays were performed with 200 mM tellurite K_2_TeO_3_ (A) or 200 mM CuSO_4_ (B) in TSB medium with (white) and without (grey) 2 mM cystine. Results represent the mean values with standard deviations and are representative of at least three independent experiments.
(0.56 MB TIF)

Table S1 Additional stress-regulated genes differentially expressed in the *S. aureus* Δ*cymR* mutant strain compared with strain SH1000.
(0.11 MB PDF)

Table S2 Relative expression levels of stress response-associated genes in *S. aureus* mutant strains compared to SH1000.
(0.09 MB PDF)

Table S3 Intracellular metabolite estimation in *S. aureus cymR* mutant strain compared to SH1000.
(0.08 MB PDF)

Table S4 Virulence or host-interaction associated genes differentially expressed in the *S. aureus* Δ*cymR* mutant strain compared to SH1000.
(0.11 MB PDF)

Table S5 Oligonucleotides used in this study.
(0.07 MB PDF)
