# Supplementary figures and images for: The Pleiotropic CymR Regulator of Staphylococcus aureus Plays an Important Role in Virulence and Stress Response
Source: PLoS Pathog. 2010 May 13;6(5):e1000894. doi: 10.1371/journal.ppat.1000894 (PMC2869319; doi:10.1371/journal.ppat.1000894)

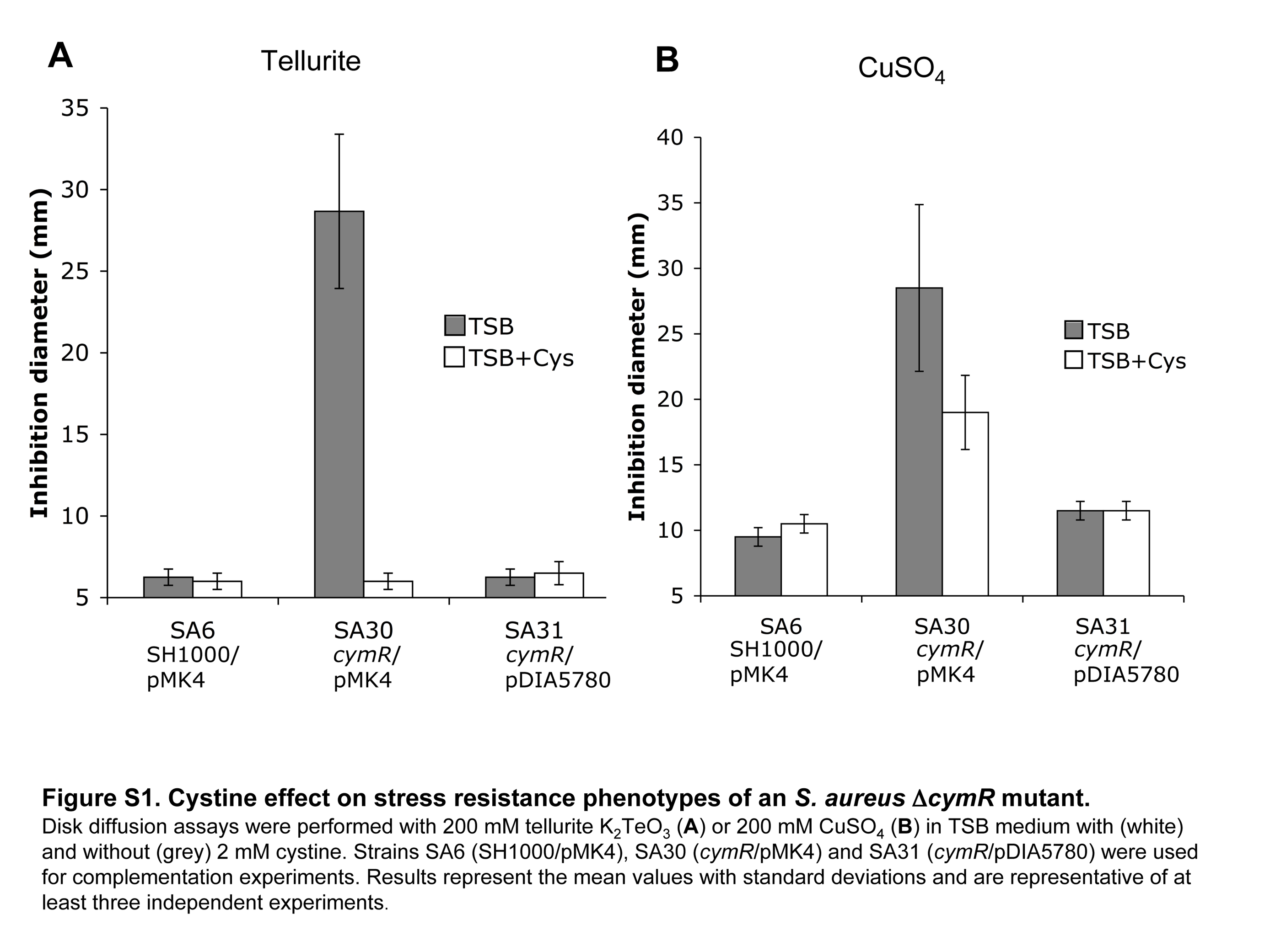

Supplement: Figure S1 — Cystine effect on stress resistance phenotypes of an S. aureus ΔcymR mutant. Disk diffusion assays were performed with 200 mM tellurite K2TeO3 (A) or 200 mM CuSO4 (B) in TSB medium with (white) and without (grey) 2 mM cystine. Strains SA6 (SH1000/pMK4), SA30 (cymR/pMK4) and SA31 (cymR/pDIA5780) were used for complementation experiments. Results represent the mean values with standard deviations and are representative of at least three independent experiments. (0.62 MB TIF) [file ppat.1000894.s001.tif]

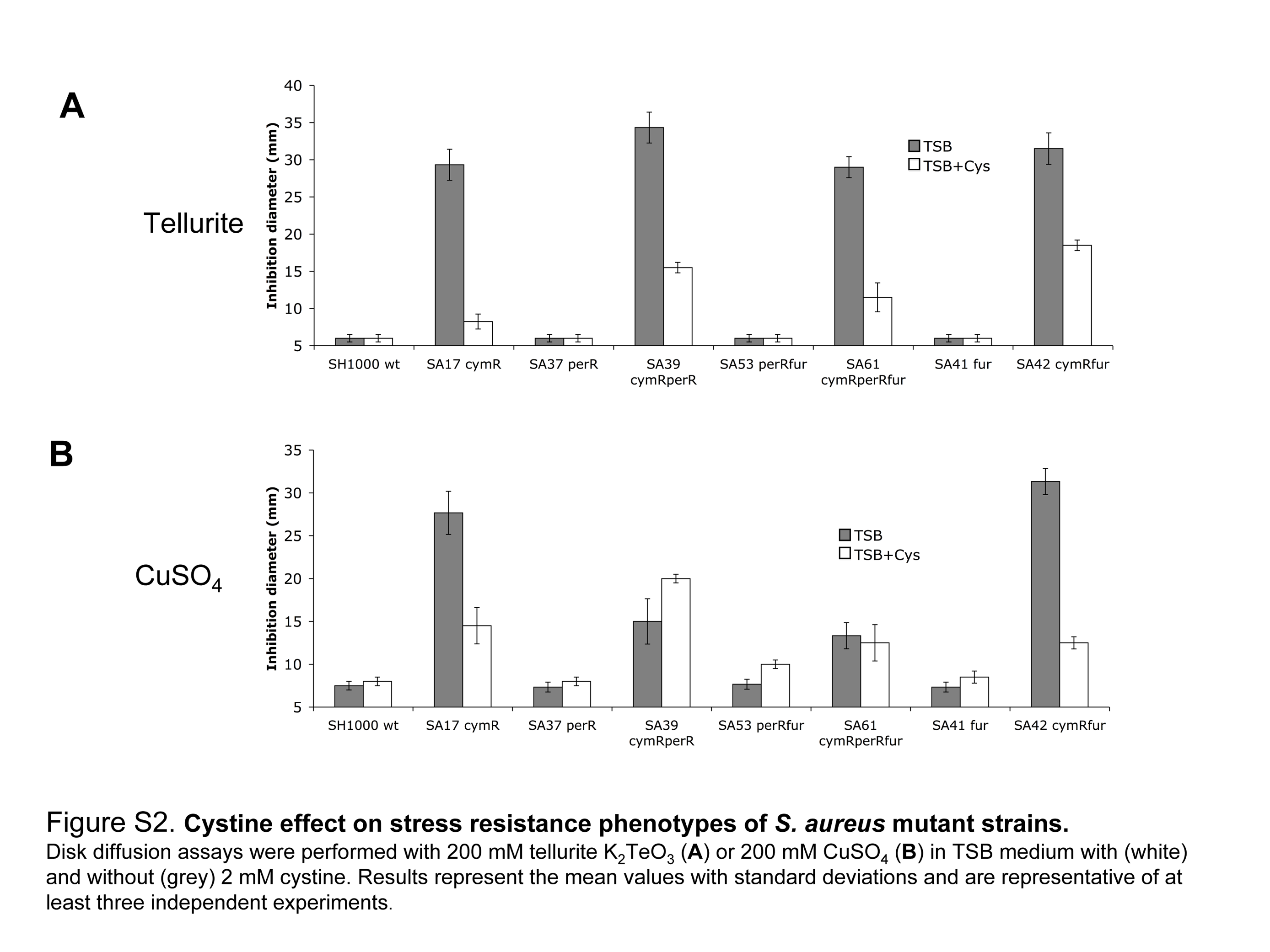

Supplement: Figure S2 — Cystine effect on stress resistance phenotypes of S. aureus mutant strains. Disk diffusion assays were performed with 200 mM tellurite K2TeO3 (A) or 200 mM CuSO4 (B) in TSB medium with (white) and without (grey) 2 mM cystine. Results represent the mean values with standard deviations and are representative of at least three independent experiments. (0.56 MB TIF) [file ppat.1000894.s002.tif]
