# Supplementary material for: The Pleiotropic CymR Regulator of Staphylococcus aureus Plays an Important Role in Virulence and Stress Response
Source: PLoS Pathog. 2010 May 13;6(5):e1000894. doi: 10.1371/journal.ppat.1000894 (PMC2869319; doi:10.1371/journal.ppat.1000894)
Supplement: Table S1 — Additional stress-regulated genes differentially expressed in the S. aureus ΔcymR mutant strain compared with strain SH1000. (0.11 MB PDF) [file ppat.1000894.s003.pdf]

Table S1. Additional stress-regulated genes differentially expressed in the *S. aureus* Δ*cymR* mutant strain compared with strain SH1000

Gene name[a] (synonym)	Function/similarity	Transcriptome analysis[b]		Differential expression under stress conditions[c]
		Δ*cymR*/ SH1000 expression ratio	*P* value	
SA1315	ferredoxin, putative	5.38	0.0129	Nitrite
SA1316	conserved hypothetical protein	2.09	<1.0E-16	H_2O_2, nitrite
SA0938	cytochrome d ubiquinol oxidase, subunit II	2.40	<1.0E-16	H_2O_2, nitrosative, nitrite
SA0937	cytochrome d ubiquinol oxidase, subunit I	2.36	<1.0E-16	H_2O_2, nitrosative, nitrite
SA1175	hypothetical protein	2.25	<1.0E-16	H_2O_2
SA0509	conserved hypothetical protein	2.19	<1.0E-16	General (sigma B), nitrite
SA1589 *ribD*	riboflavin biosynthesis RibD	2.01	0.0129	H_2O_2
SA0517	hydrolase, haloacid dehalogenase-like family	1.91	2.22E-16	Nitrite
SA1691	transglycosylase domain protein	1.83	1.18E-12	Nitrite
SA0817	FMN oxidoreductase	1.76	7.92E-06	H_2O_2, nitrite
SA0800	conserved hypothetical protein	1.72	1.00E-07	H_2O_2
SA0205	M23/M37 peptidase domain protein	1.67	3.81E-09	H_2O_2, nitrite
SA1588 *ribE*	riboflavin synthase, alpha subunit	1.64	0.0129	H_2O_2
SA1989*	alcohol dehydrogenase, zinc-containing	1.64	1.71E-06	Nitrite
SA1235	putative DNA binding protein	1.63	1.54E-08	Nitrite
SA2328	conserved hypothetical protein	1.60	0.00018	General (sigma B), H_2O_2
SA1174 *lexA*	LexA repressor	1.57	1.70E-07	H_2O_2
SA0774	ABC transporter, ATP-binding protein	1.54	6.46E-07	General (sigma B)
SA1710	exonuclease	1.52	1.30E-06	H_2O_2, nitrosative, nitrite
SA0526	conserved hypothetical protein	1.51	8.32E-05	Nitrite
SA2328	membrane protein, putative, authentic frameshift	1.50	3.33E-09	H_2O_2
SA0857 *mecA**	negative regulator of competence	1.50	3.37E-06	H_2O_2, nitrite
SA2490	N-acetyltransferase family protein	1.49	4.29E-06	Nitrite
SA1149 *glnR*	glutamine synthetase repressor	1.49	0.05	H_2O_2, nitrosative
SA0524	conserved hypothetical protein TIGR00294	1.47	7.29E-07	Nitrite
SA1328 *xerD**	site-specific recombinase XerD	1.46	1.07E-05	Nitrite
SA1150 *glnA**	FemC protein, glutamine synthetase	1.45	0.0129	H_2O_2, nitrosative
SA1128*	RecA protein	1.44	2.23E-05	H_2O_2, nitrite
SA2142*	drug resistance transporter, EmrB/QacA subfamily	1.40	0.001	H_2O_2, nitrite
SA0412	conserved hypothetical protein	1.35	6.14E-07	H_2O_2
SA2322	conserved hypothetical protein	0.71	0.00013	H_2O_2
SA0747 *cspC*	cold-shock protein CspC	0.71	0.0089	Nitrite
SA1548	1-acyl-sn-glycerol-3-phosphate acyltransferase, putative	0.71	0.0032	H_2O_2
SA2485*	hypothetical protein	0.70	8.92E-05	General (sigma B)
SA1140 *glpF**	glycerol uptake facilitator protein	0.70	8.78E-05	H_2O_2
SA2197	conserved hypothetical protein	0.68	2.46E-06	H_2O_2
SA0873*	conserved hypothetical protein	0.67	6.85E-06	Nitrite

SA2374	conserved hypothetical protein	0.66	5.02E-06	General (sigma B), H_2O_2
SA1547*	PTS system, N-acetylglucosamine-specific component	0.65	6.25E-06	H_2O_2, nitrite
SA0617*	transporter, putative	0.63	0.01	H_2O_2
SA0479		0.61	4.29E-08	H_2O_2
SA0360*	conserved hypothetical protein	0.60	9.96E-06	General (sigma B), H_2O_2
SA0341*	hypothetical protein	0.60	0.00018	H_2O_2, nitrite
SA2098	D-isomer specific 2-hydroxyacid dehydrogenase family protein	0.57	4.22E-10	Nitrite
SA0359	conserved hypothetical protein	0.57	0.005	H_2O_2, nitrite
SA2102*	formate dehydrogenase, alpha subunit, putative	0.55	4.26E-11	General (sigma B)
SA0996 *sdhB**	succinate dehydrogenase, iron-sulfur protein	0.53	4.41E-09	Nitrosative, nitrite
SA0995 *sdhA**	succinate dehydrogenase, flavoprotein subunit	0.51	3.46E-14	Nitrosative, nitrite
SA0682*	proton-dependent oligopeptide transporter family protein	0.51	1.23E-13	H_2O_2
SA0131 *deoD**	purine nucleoside phosphorylase	0.29	0.0052	H_2O_2

a. The SA numbers (N315 strain) for *S. aureus* genes correspond to those of AureoList (http://genolist.pasteur.fr/AureoList/).
"*" indicates genes differentially expressed upon internalization of *S. aureus* in human epithelial cells [1].
b. The results obtained are representative of 8 hybridizations from 4 independent cultures in TSB medium with 2 mM cystine. The data sets generated were loaded into the GenoScript Database (http://genoscript.pasteur.fr)[2].
c. Differential expression of corresponding genes under indicated stress conditions including hydrogen peroxide (H_2O_2) [3], nitrite [4], nitrosative [5] and general (sigma B) [6] stresses.

References:

1. Garzoni C, Francois P, Huyghe A, Couzinet S, Tapparel C, et al. (2007) A global view of *Staphylococcus aureus* whole genome expression upon internalization in human epithelial cells. BMC Genomics 8: 171.
2. Soutourina O, Poupel O, Coppee JY, Danchin A, Msadek T, et al. (2009) CymR, the master regulator of cysteine metabolism in *Staphylococcus aureus*, controls host sulfur source utilization and plays a role in biofilm formation. Mol Microbiol 73: 194-211.
3. Chang W, Small DA, Toghrol F, Bentley WE (2006) Global transcriptome analysis of *Staphylococcus aureus* response to hydrogen peroxide. J Bacteriol 188: 1648-1659.
4. Schlag S, Nerz C, Birkenstock TA, Altenberend F, Gotz F (2007) Inhibition of staphylococcal biofilm formation by nitrite. J Bacteriol 189: 7911-7919.
5. Richardson AR, Dunman PM, Fang FC (2006) The nitrosative stress response of *Staphylococcus aureus* is required for resistance to innate immunity. Mol Microbiol 61: 927-939.
6. Bischoff M, Dunman P, Kormanec J, Macapagal D, Murphy E, et al. (2004) Microarray-based analysis of the *Staphylococcus aureus* sigmaB regulon. J Bacteriol 186: 4085-4099.
