# Supplementary material for: The Pleiotropic CymR Regulator of Staphylococcus aureus Plays an Important Role in Virulence and Stress Response
Source: PLoS Pathog. 2010 May 13;6(5):e1000894. doi: 10.1371/journal.ppat.1000894 (PMC2869319; doi:10.1371/journal.ppat.1000894)
Supplement: Table S2 — Relative expression levels of stress response-associated genes in S. aureus mutant strains compared to SH1000. (0.09 MB PDF) [file ppat.1000894.s004.pdf]

Table S2. Relative expression levels of stress response-associated genes in *S. aureus* mutant strains compared to SH1000.

Gene name	**H_2O_2/stressless conditions expression ratio**		**cymR mutant/SH1000 expression ratio**						
	SH1000	*cymR*	*cymR*	*perR*	*cymRperR*	*perRfur*	*cymRperRfur*	*fur*	*cymRfur*
ahpF	**7.8**	0.97	**12.01**	**28.03**	**15.06**	**26.87**	**6.62**	**17.41**	**13.15**
dps	**22.8**	**9.29**	**4.40**	**82.71**	**13.14**	**92.52**	**4.85**	**2.67**	**6.89**
sodA	0.98	0.54	**5.05**	1.08	**5.89**	1.36	**3.64**	1.44	**5.05**
sodM	0.59	0.81	**2.86**	1.15	**3.53**	1.78	**2.08**	1.74	**4.44**
copA	0.72	0.86	**11.83**	1.09	**26.14**	0.97	**10.29**	1.26	**13.11**

For qRT-PCR analysis, total RNA was extracted from *S. aureus* strains grown in TSB medium supplemented with 2 mM cystine. For H_2O_2 stress induction the bacteria were incubated with 20 mM H_2O_2 for 10 minutes. After reverse transcription, specific cDNAs were quantified by qRT-PCR using 16S rRNA gene for normalization. In bold are indicated the expression ratios > 2 of regulated genes.
