# Supplementary material for: The Pleiotropic CymR Regulator of Staphylococcus aureus Plays an Important Role in Virulence and Stress Response
Source: PLoS Pathog. 2010 May 13;6(5):e1000894. doi: 10.1371/journal.ppat.1000894 (PMC2869319; doi:10.1371/journal.ppat.1000894)
Supplement: Table S3 — Intracellular metabolite estimation in S. aureus cymR mutant strain compared to SH1000. (0.08 MB PDF) [file ppat.1000894.s005.pdf]

Table S3. Intracellular metabolite estimation in *S. aureus cymR* mutant strain compared to SH1000.

**Strain**<br>**Metabolite**	**SH1000**	**SA17 *cymR***	***cymR*/SH1000 ratio**
Aspartate	4785	6298	1.3
Glutamate	6587	1617	0.25
Cysteine	18.5	1270	68
Proline	1674	1900	1.1
Glycine	337	253.5	0.75
Alanine	935	968	1
Valine	341	268	0.8
Cystine	37	71	1.9
Homocysteine	15.5	91	5.8
Methionine	32	52	1.60
Cystathionine	15.5	29	1.85
Isoleucine	182	149	0.8
Leucine	809	673	0.8
Tyrosine	45	57	1.3
Phenylalanine	200	177	0.9
NH_3	373	384	1
Lysine	277	279	1
Histidine	31	36	1.1

Intracellular metabolite concentrations given in µM are mean values of at least 4 independent experiments with *S. aureus* strains grown in TSB medium supplemented with 2 mM cystine. Standard deviations were less than 15% of the mean.
