# Supplementary material for: The Pleiotropic CymR Regulator of Staphylococcus aureus Plays an Important Role in Virulence and Stress Response
Source: PLoS Pathog. 2010 May 13;6(5):e1000894. doi: 10.1371/journal.ppat.1000894 (PMC2869319; doi:10.1371/journal.ppat.1000894)
Supplement: Table S4 — Virulence or host-interaction associated genes differentially expressed in the S. aureus ΔcymR mutant strain compared to SH1000. (0.11 MB PDF) [file ppat.1000894.s006.pdf]

Table S4. Virulence or host-interaction associated genes differentially expressed in the *S. aureus* Δ*cymR* mutant strain compared to SH1000

**Gene name**[a] (synonym)	**Function/similarity**	**Transcriptome analysis**[b]		**qRT-PCR**[c]	**Differential expression in strains mutant for virulence regulators**[d]
		Δ*cymR*/ SH1000 expression ratio	*P* value	Δ*cymR*/ SH1000 expression ratio	
SA0363*	conserved hypothetical protein	0.18	0.00025		SarA
SA2353*	ssaA homolog	0.47	<1.0E-16	0.44	WalKR
SA0271	conserved hypothetical protein	0.50	1.71E-09		SarA, Rot, AgrA
SA1898	SceD protein, putative	0.51	2.97E-13	0.41	WalKR
SA0650	drug transporter	0.51	1.04E-13		MgrA
SA2343	hypothetical protein	0.51	1.87E-12		AgrA, SarA, MgrA
SA0675	ABC transporter, ATP-binding protein	0.51	0.025		Rot
SA2378*	glyoxalase family protein	0.54	1.01E-11	0.48	AgrA, SarA, MgrA, Rot
SA2082 *ureA*	urease, gamma subunit	0.54	1.03E-11		Rot, ArlR, MgrA
SA1747*	ABC transporter, ATP-binding protein	0.55	1.42E-06		SarA
SA2239	amino acid permease	0.57	5.50E-09		AgrA, MgrA
SA0443	conserved hypothetical protein	0.58	1.89E-09		MgrA
SA0432*	PTS system component	0.58	2.65E-09		AgrA
SA1965 *femD**	phosphoglucosamine mutase	0.60	8.24E-09		
SA0672*	conserved hypothetical protein TIGR00370	0.60	9.69E-08		SarA, SaeSR
SA0244 *tagF*	teichoic acid biosynthesis protein F	0.63	3.09E-07	0.52	SarA
SA0523	glycosyl transferase, group 1	0.64	1.45E-06		SarA, Rot
SA0423	LysM domain protein	0.64	0.033		SarA
SA1985*	hypothetical protein	0.64	0.0019		SarA
SA0796 *dltD**	extramembranal protein	0.65	1.08E-08	0.47	
SA2261	transporter, putative	0.65	2.58E-06		Rot
SA1956*	lytic regulatory protein	0.65	1.57E-05		MgrA, SarA
SA2171	hypothetical protein	0.66	3.66E-07		Rot
SA2088 *ureD**	urease accessory protein UreD	0.66	3.75E-06		ArlSR, MgrA, Rot
SA2086 *ureF*	urease accessory protein UreF	0.67	9.48E-06		ArlSR, MgrA, Rot
SA2084 *ureC*	urease, alpha subunit	0.67	1.05E-05		ArlSR, MgrA, Rot
SA2331	hypothetical protein	0.67	1.29E-05		SarA
SA0905 *atl**	bifunctional autolysin precursor	0.67	9.78E-06		WalKR
SA0793 *dltA**	D-alanine-D-alanyl carrier protein ligase	0.68	0.044	0.51	
SA2441	glycosyl transferase, group 1	0.68	1.99E-05		
SA0794 *dltB**	DltB protein	0.69	0.0028		
SA0651*	hypothetical protein	0.70	0.00016		Rot
SA1684*	conserved hypothetical protein	1.41	5.25E-05		AgrA, SarA
SA1194 *msrA*	methionine sulfoxide reductase	1.41	0.0011		Rot
SA2007	alpha-acetolactate decarboxylase, putative	1.44	1.28E-05		AgrA, ArlSR, MgrA, Rot
SA2006*	Map protein, putative	1.54	6.37E-09		AgrA, SarA, ArlR
SA1041 *pyrR*	pyrimidine operon regulatory protein	1.56	0.012		SarA, AgrA, MgrA
SACOL0673	hypothetical protein	1.62	2.17E-08		AgrA, SarA

SACOL1186	antibacterial protein	1.64	<1.0E-16		AgrA, SarA
SA1811	phospholipase C precursor	1.69	1.35E-05		SaeR
SA1131*	pyruvate ferredoxin oxidoreductase, alpha subunit, putative	1.77	1.40E-12		Rot
SACOL1187	antibacterial protein, phenol soluble modulin	2.07	8.88E-16	2.23	SarA
SA0914	chitinase-related protein	2.15	<1.0E-16		SarA, Rot
SA0395	conserved hypothetical protein	2.15	<1.0E-16		SarA, SaeSR
SA1490	YhjN protein; toxin production and resistance	2.21	<1.0E-16	3.77	SarA
SA0395	hypothetical protein, pathogen island	2.63	<1.0E-16	5.26	ArlSR, SarA, SaeSR
SAS016*	hypothetical protein	3.87	<1.0E-16		SarA

a. The gene numbers SACOL and SA correspond to *S. aureus* COL and N315 strain genomic sequence, respectively (http://cmr.tigr.org/tigr-scripts/CMR/GenomePage.cgi?database=gsa).
"*" indicates genes differentially expressed upon internalization of *S. aureus* in human epithelial cells [1].
b. The results obtained are representative of 8 hybridizations from 4 independent cultures. The data sets generated were loaded into the GenoScript Database (http://genoscript.pasteur.fr)[2].
c. For qRT-PCR analysis, total RNA was extracted from *S. aureus* strains grown in TSB medium supplemented with 2 mM cystine.
d. Differential expression of corresponding genes in strains deficient for the indicated virulence regulators, including SarA [3-5], AgrA [3,4], ArlSR [6], SaeSR [7], Rot [8], MgrA [9] and WalKR [10].

References:

1. Garzoni C, Francois P, Huyghe A, Couzinet S, Tapparel C, et al. (2007) A global view of *Staphylococcus aureus* whole genome expression upon internalization in human epithelial cells. BMC Genomics 8: 171.
2. Soutourina O, Poupel O, Coppee JY, Danchin A, Msadek T, et al. (2009) CymR, the master regulator of cysteine metabolism in *Staphylococcus aureus*, controls host sulfur source utilization and plays a role in biofilm formation. Mol Microbiol 73: 194-211.
3. Cassat J, Dunman PM, Murphy E, Projan SJ, Beenken KE, et al. (2006) Transcriptional profiling of a *Staphylococcus aureus* clinical isolate and its isogenic *agr* and *sarA* mutants reveals global differences in comparison to the laboratory strain RN6390. Microbiology 152: 3075-3090.
4. Dunman PM, Murphy E, Haney S, Palacios D, Tucker-Kellogg G, et al. (2001) Transcription profiling-based identification of *Staphylococcus aureus* genes regulated by the *agr* and/or *sarA* loci. J Bacteriol 183: 7341-7353.
5. Nagarajan V, Smeltzer MS, Elasri MO (2009) Genome-scale transcriptional profiling in *Staphylococcus aureus* : bringing order out of chaos. FEMS Microbiol Lett.
6. Liang X, Zheng L, Landwehr C, Lunsford D, Holmes D, et al. (2005) Global regulation of gene expression by ArlRS, a two-component signal transduction regulatory system of *Staphylococcus aureus*. J Bacteriol 187: 5486-5492.
7. Rogasch K, Ruhmling V, Pane-Farre J, Hoper D, Weinberg C, et al. (2006) Influence of the two-component system SaeRS on global gene expression in two different *Staphylococcus aureus* strains. J Bacteriol 188: 7742-7758.
8. Said-Salim B, Dunman PM, McAleese FM, Macapagal D, Murphy E, et al. (2003) Global regulation of *Staphylococcus aureus* genes by Rot. J Bacteriol 185: 610-619.
9. Luong TT, Dunman PM, Murphy E, Projan SJ, Lee CY (2006) Transcription Profiling of the *mgrA* Regulon in *Staphylococcus aureus*. J Bacteriol 188: 1899-1910.
10. Dubrac S, Boneca IG, Poupel O, Msadek T (2007) New insights into the WalK/WalR (YycG/YycF) essential signal transduction pathway reveal a major role in controlling cell wall metabolism and biofilm formation in *Staphylococcus aureus*. J Bacteriol 189: 8257-8269.
