# Supplementary material for: The Pleiotropic CymR Regulator of Staphylococcus aureus Plays an Important Role in Virulence and Stress Response
Source: PLoS Pathog. 2010 May 13;6(5):e1000894. doi: 10.1371/journal.ppat.1000894 (PMC2869319; doi:10.1371/journal.ppat.1000894)
Supplement: Table S5 — Oligonucleotides used in this study. (0.07 MB PDF) [file ppat.1000894.s007.pdf]

Table S5. Oligonucleotides used in this study

Name	Description	Sequence (5’-3’)
OS66	5' 16SrRNA (qRT-PCR reference gene)	ACGTGGATAACCTACCTATAAGACTGGGAT
OS67	3' 16SrRNA	TACCTTACCAACTAGCTAATGCAGCG
OS102	5'-*ahpF* (qRT-PCR)	AGGTTAGTGGTCGTGCACCT
OS103	3'-*ahpF*	GCTTGAACGACATCAGGACA
OS104	5'-*copA* (qRT-PCR)	CGGTGTAAATGATGCACCTG
OS105	3'-*copA*	ATCAAGTCGCCACCAAGAAT
OS106	5'-*dps* (qRT-PCR)	TGTTAAAGAAGCTGCGAAAGG
OS107	3'-*dps*	AGCAATTTCGATTGCGTTTT
OS108	5'-*sodA* (qRT-PCR)	GCGGTGGACATTTAAACCAT
OS109	3'-*sodA*	CAGCTGCTTTGTCAGCAAAT
OS114	5'-*trxB* (qRT-PCR)	AACCATTAACAGCGCCATTT
OS115	3'-*trxB*	AACCTTTGTCGCGAACATCT
OS116	5'-*trxA* (qRT-PCR)	TAGATTTTTGGGCAACATGG
OS117	3'-*trxA*	AGCTGCAGTTGATGGATTTTC
OS118	5'-*sodM* (qRT-PCR)	CGCAACAGTTGAAGGAACAG
OS119	3'-*sodM*	CACCGCCATTATTACGGACT
OS122	5'-*fur* (qRT-PCR)	GATAAAGCGCCTGAAATTGG
OS123	3'-*fur*	TGTTTTGCGCCTTCTTTTCT
OS128	5'-*perR* (qRT-PCR)	AATTGCATCATTGCGACAAG
OS129	3'-*perR*	TTTCATCAGCTGTTGGATGAGT
OS144	5'-*copA* (gel-shift, 316 bp)	CCTAAGAATTGCAAATCCAGAAGT
OS145	3'-*copA*	CAGTTTCTTTTCGATACGATTTGA
OS146	5'-*perR* (gel-shift, 346 bp)	TTTGAAAATGAACCTTTGAAACCT
OS147	3'-*perR*	GCAATGATGCAATTGATTCTTCTA
OS148	5'-*fur* (gel-shift, 324 bp)	TATGTATGGTTCACCAGGTTTTTG
OS149	3'-*fur*	CGATGTCCACTCCCCTACTAATAA
OS150	5'-*ahpC* (gel-shift, 331 bp)	AAGGTTGACTTAGTTGAGAATACAAAT
OS151	3'-*ahpC*	CGCTGTAAATGGTAAGATTTCTTTG
OS152	5'-*sodA* (gel-shift, 347 bp)	AAGTTTATGGTGTATGTGAGTCTTGC
OS153	3'-*sodA*	CAAATGCGTATGGTAATTTTGGTA
OS154	5'-*dps* (gel-shift, 352 bp)	ATGTGGAAAACAACATGGCACCA
OS155	3'-*dps*	GTAAGCTACTGTCCAGTTTGCTA
OS85	5'-*tcyP* (gel-shift, 293 bp)	CCTGCTTCATTTTCAAATATTAACT
OS86	3'-*tcyP*	GCAATATTACGATGAAAATCACCA
